# Immune Responses to ESAT-6 and CFP-10 by *FASCIA* and Multiplex Technology for Diagnosis of *M. tuberculosis* Infection; IP-10 Is a Promising Marker

**DOI:** 10.1371/journal.pone.0043438

**Published:** 2012-11-08

**Authors:** Emilie Borgström, Peter Andersen, Fredrik Atterfelt, Inger Julander, Gunilla Källenius, Markus Maeurer, Ida Rosenkrands, Maria Widfeldt, Judith Bruchfeld, Hans Gaines

**Affiliations:** 1 Unit of Infectious Diseases, Institution of Medicine, Karolinska Institutet, Karolinska University Hospital, Solna, Stockholm, Sweden; 2 Statens Serum Institut, Copenhagen S, Denmark; 3 Swedish Institute for Communicable Disease Control, Solna, Stockholm, Sweden; 4 Karolinska Institutet, Department of Clinical Science and Education, Södersjukhuset, Stockholm, Sweden; 5 Microbiology and Tumor Biology Center, Karolinska Institute, Stockholm, Sweden; National Institute for Infectious Diseases (L. Spallanzani), Italy

## Abstract

**Background:**

There is a need for reliable markers to diagnose active and latent tuberculosis (TB). The interferon gamma release assays (IGRAs) are compared to the tuberculin skin test (TST) more specific, but cannot discriminate between recent or remote TB infection. Here the Flow-cytometric Assay for Specific Cell-mediated Immune-response in Activated whole blood (*FASCIA*), which quantifies expanded T-lymphoblasts by flow-cytometric analysis after long-term antigen stimulation of whole blood, is combined with cytokine/chemokine analysis in the supernatant by multiplex technology for diagnosis of *Mycobacterium tuberculosis (Mtb)* infection.

**Methods and Findings:**

Consecutive patients with suspected TB (n = 85), with microbiologically verified active pulmonary TB (n = 33), extra pulmonary TB (n = 21), clinical TB (n = 11), presumed latent TB infection (LTBI) (n = 23), patients negative for TB (n = 8) and 21 healthy controls were studied. Blood samples were analyzed with FASCIA and multiplex technology to determine and correlate proliferative responses and the value of 14 cytokines for diagnosis of *Mtb* infection: IFN- γ, IL-2, TNF-α, IP-10, IL-12, IL-6, IL-4, IL-5, IL-13, IL-17, MIP-1β, GM-CSF, IFN-α2 and IL-10. Cytokine levels for IFN-γ, IP-10, MIP-1β, IL-2, TNF-α, IL-6, IL-10, IL-13 and GM-CSF were significantly higher after stimulation with the *Mtb* specific antigens ESAT-6 and CFP-10 in patients with active TB compared to healthy controls (p<0.05) and correlated with proliferative responses. IP-10 was positive in all patients with verified TB, if using a combination of ESAT-6 and CFP-10 and was the only marker significantly more sensitive in detecting active TB then IFN-γ (p = 0.012). Cytokine responses in patients with active TB were more frequent and detected at higher levels than in patients with LTBI.

**Conclusions:**

IP-10 seems to be an important marker for diagnosis of active and latent TB. Patients with active TB and LTBI responded with similar cytokine profiles against TB antigens but proliferative and cytokine responses were generally higher in patients with active TB.

## Introduction

An estimated one-third of the world's population is possibly infected with *Mycobacterium tuberculosis* (*Mtb*) [1] and thereby constitutes a source of latent tuberculosis infection (LTBI) that can lead to symptomatic, often contagious, tuberculosis (TB). Several methods are available for detecting tubercle bacilli in active TB. In immune-deficient patients, e.g. HIV-infected, and in children, rapid diagnostic methods such as the traditional smear for detection of acid fast bacilli and *Mtb complex* PCR, as well as the more sensitive but inherently slower mycobacterial culture, often fail [2] and new diagnostic tools are needed. For LTBI diagnosis there is no golden standard [3]. The tuberculin skin test (TST) [4] and the more specific [5] interferon gamma release assays (IGRA) [6], [7] are indirect diagnostic methods for LTBI, but cannot discriminate between active TB or different clinical entities of LTBI, such as *Mtb* infection, or a mere immunological memory of previous TB disease [8], [9], [10]. An important component in TB control in low TB endemic areas is the detection of recently TB infected individuals, in particular those with an increased risk of progressing to active disease [3], [9]. The need to find new antigens and immunological markers for this patient category is urgent, since IGRAs are not convincingly better at predicting this condition than the TST [11], [12]. With adequate prophylactic treatment, patients with LTBI will not develop symptomatic disease and the chain of transmission can thus be broken [13].

Other immunological markers than interferon-gamma (IFN-γ), such as interleukin-2 (IL-2), interferon-inducible protein-10 (IP-10) and monocyte chemotactic protein 2 (MCP-2), have been suggested as more sensitive for detecting active TB [14], [15], [16] and LTBI [17], [18]. Some studies show differences in cytokine profiles between active and LTBI [19], [20], [21], but this is a new area of research and further studies are needed to confirm the results.

Our aim with the present study was to evaluate cytokine profiles influencing the balance between the effector and suppressive immune responses that seem to be essential for affecting the clinical outcome of mycobacterial infection [22]. Another aim was to investigate possible biomarkers for *Mtb* infection. Blood samples from patients with active TB and controls were analysed using Flow-cytometric Assay for Specific Cell-mediated Immune-response in Activated whole blood (*FASCIA*) after long-term stimulation with specific *Mtb* antigens [23], [24], [25], [26,] in combination with cytokine/chemokine analysis in the supernatant by multiplex technology. Diluted whole-blood cultures used for FASCIA allow long-term periods of culture, thus enabling the detection of responses which may not reach a level of detection when cells are only cultured over-night, which in general is the case for other methods such as intra-cellular staining, ELISpot and the commercially available IGRA tests Quantiferon and T-SPOT.TB. During long-term cultures, specific cells divide repeatedly and the number of specific responding cells is multiplied resulting in increasing concentrations of the cytokines produced. We have evaluated different periods of culture and determined appropriate periods, such as 3 or 7 days, for different cytokines to reach peak levels (25). The benefit of being able to examine in great detail different cytokine profiles of responding cells is that it may elucidate the nature of the immune response and provide insights into TB immunopathology. This may be particularly important for the study of TB immune response where negative vs. positive is not always the only question to be asked. A more useful tool may be one that can accurately distinguish between responses against TB-antigens in individuals with latent vs. active TB infection; or with LTBI well controlled vs. infection that will probably be activated soon; or with LTBI that may be activated vs. a state of post-infection when no viable *Mtb* can be activated to replicate – following treatment or killing the bacilli by the host.

In this study, the combination of *FASCIA* and multiplex assay was assessed for the diagnosis of TB in patients with suspected active TB disease. Patients with microbiologically verified disease were used as positive controls and healthy subjects with no exposure or risk factor for TB were used as negative controls to determine a cut-off level for several cytokines and chemokines: IFN-γ, IL-2, tumor necrosis factor alpha (TNF-α), IP-10, IL-12, IL-6, IL-4, IL-5, IL-13, IL-17, macrophage inflammatory protein 1 beta (MIP-1β), granulocyte macrophage-colony stimulating factor (GM-CSF), interferon-alfa 2 (IFN-α2) and IL-10. They were also used to calculate the assay's sensitivity and specificity for the diagnosis of *Mtb* infection. The biomarkers chosen are all probably involved in the innate or adaptive immune response to *Mtb*, such as leukocyte activation and granuloma formation. IFN-γ activates both macrophages and CD4+ T-cells, playing a key role in initiating mycobacterial immunity [27], [28]. The other cytokines and chemokines were included to examine their suggested involvement in the differentiation of CD3+CD4+ cells into Th1 pro-inflammatory responses (IFN-γ, IL-2, TNF-α2, IP-10, IL-12 and IL-6), or Th2- (IL-4, IL-5, IL-13), Th17- (IL-17) and T- regulatory responses (IL-10) [29], [30] in *Mtb* infection.

## Materials and Methods

### 1 Ethics statement

The Regional Ethical Review Board at the Karolinska Institute in Stockholm approved of the study. Patients were included after giving their verbal and written informed consent when the nature and possible consequences of the study had been fully explained. Laboratory samples were coded and analysed in a blinded fashion.

### 2 Participants and procedures

This study was performed on samples used by us in a previous study [31]. Consecutive patients referred to the TB centre at the Clinic of Infectious Diseases at Karolinska University Hospital, Stockholm, Sweden, were recruited to the study between 2006-11-07 and 2008-03-12 after verbal and written informed consent.

The inclusion criterion was the attending physician's sufficient suspicion of active TB to warrant taking a specimen for mycobacterial analysis, including microscopy for acid-fast bacilli, *Mtb* complex polymerase chain reaction (PCR) and mycobacterial culture. The exclusion criterion was ongoing TB treatment for more than one week. Patients were examined with standardized questionnaires regarding their clinical status with symptom screening, including cough, fever, haemoptysis and weight loss, bacille Calmette-Guerin vaccine (BCG) status, age, gender, origin and immune-suppression. In addition, TB investigation was performed according to clinical routines, with radiology and 3 sputum samples for smear microscopy, polymerase chain reaction for the *Mtb* complex and mycobacterial culture. Bronchoscopy and histopathological examination of tissue biopsies were performed when appropriate. In most cases the TST was performed and read before *FASCIA* testing with the Mantoux method by nurses specifically trained for the technique. Two units of purified protein derivative (Tuberculin PPD RT23, Statens Serum Institut, Copenhagen, Denmark) were injected intradermally and cutaneous induration was measured with a ruler after 72 hours. A positive TST was defined as an induration of 10 mm or more and TST conversion as a test converting from negative to positive with an increase in size of at least 10 mm. Venous blood samples were drawn and then coded and analysed in a blinded fashion. For *FASCIA*, 5 ml blood was collected in a sodium heparin tube.

#### Case definitions

a) and b) Verified active pulmonary TB (PTB) and extra pulmonary TB (EPTB), respectively: patients with suggestive clinical symptoms and microbiological verification by either smear microscopy, *Mtb* PCR, mycobacterial culture or histopathological diagnosis of biopsy material from lymph nodes indicating granulomatous lymphadenitis. c) Clinical TB: patients with suggestive clinical symptoms, suspicious radiology and recovery after TB treatment, but without microbiological verification of the disease. d) LTBI: patients with TST ≥10 mm and with exposure to TB disease or originating from a TB high endemic country (TB incidence >100/100 000) (http://www.who.int/en/) but without radiological evidence of ongoing or previous active TB in the clinical work-up. Patients who did not receive treatment for LTBI were followed with a clinical examination and chest X-ray every 6 months for a total of two years after the initial evaluation. e) No TB: patients with TST <10 mm, no exposure to TB, no history of earlier active TB disease, negative TB investigation and some other explanation of symptoms presented (see results).

Of an initial 161 patients, 85 were analyzed with multiplex assay. All patients from the initial study with active TB (confirmed or suspected) [31] and all patients who were negative for TB in the diagnostic work up were included. In addition, patients with a positive TST from high-endemic countries were chosen to represent LTBI and patients with a recent exposure to active TB and sometimes a TST conversion were chosen to represent recent LTBI. These two groups of LTBI were later analyzed together since there were no differences found between them in the cytokine analysis.

Of the 85 patients included for multiplex analysis, 25 had verified PTB, 18 verified EPTB, 11 non-verified TB patients had clinical TB, 23 had LTBI and 8 were TB negative ( Table 1).

**Table 1:** Demographic and clinical data in 85 patients investigated for TB.

Patient Category	Female gender n(%)	Age median (range)	BCG n(%)	Origin n(%) Low-High- endemicendemic	Immune-suppressionn (%)	HIV+/HIV tested n(%)	Culture+or PCR+ n(%)	TST+/TST tested n(%)
Pulm TB n = 25	12 (48)	34 (17–66)	17 (68)	9 (36)16 (64)	7 (20)	2/19 (11)	25 (100)	14/16 (93)
ExpulmTB n = 18	13 (72)	39 (19–67)	8 (44)	4 (22)14 (78)	3 (17)	0/14 (0)	15 (83)	12/12 (100)
Clinical TB n = 11	8 (73)	43 (20–79)	5(45)	8 (73)3 (27)	5 (45)	0/6(0)	0 (0)	7/8 (88)
Non TBn = 8	5 (63)	47 (23–76)	5 (63)	4 (50)4 (50)	0 (0)	0/4 (0)	-	0/11 (0)
Latent TB n = 23	11 (48)	44 (20–67)	18 (78)	14 (61)9 (39)	6 (26)	0/5 (0)	-	23/23 (100)

Values given are the number of patients in each category (percentage in parentheses). Female gender is given. Low TB-endemic origin incidence ≤1/100000, High TB-endemic origin incidence >100/100000. Immunosuppression includes potentially immunosuppressive conditions such as diabetes mellitus, treatment with immunosuppressant drugs, transplantation, renal insufficiency, rheumatic diseases, alcoholism and pregnancy.

#### Controls

Students (n = 17) and laboratory personnel (n = 4) were consecutively recruited to the study as healthy controls after verbal and written informed consent. Inclusion criteria were no previous TB history, no contact with a TB patient, no stay in a TB endemic country for more than 3 months (outside Western Europe, North America or Australia), no environmental mycobacterial infection and not worked more than one year in a hospital, a prison, with asylum-seekers or in similar institutions. Flow cytometry was run in all 21 controls, but multiplex assay was only run in 17 (day 7) and 15 (day 3) samples due to loss of samples. The results were used to establish cut-off levels for positive cytokine reactions with the multiplex assay.

### 3 Laboratory procedures

Smear microscopy for acid-fast bacilli by auramine-fluorescence staining was done after concentration, polymerase chain reaction for the *M tb* complex using the COBAS AMPLICOR MTB test (Roche, Branchburg, NJ, USA) and mycobacterial culture with the BACTEC 960 MGIT system (BD, Sparks, MD, USA) and on conventional Löwenstein-Jensen media.

In patients with verified TB, human immunodeficiency virus (HIV) testing was performed at the Microbiology Department, Karolinska Hospital, with chemiluminescence detection of both antibodies and viral antigens, using Architect HIV Combo (Abbott Scandinavia AB, Stockholm, Sweden).

#### Cell-mediated immune responses by FASCIA

**Figure 1:**
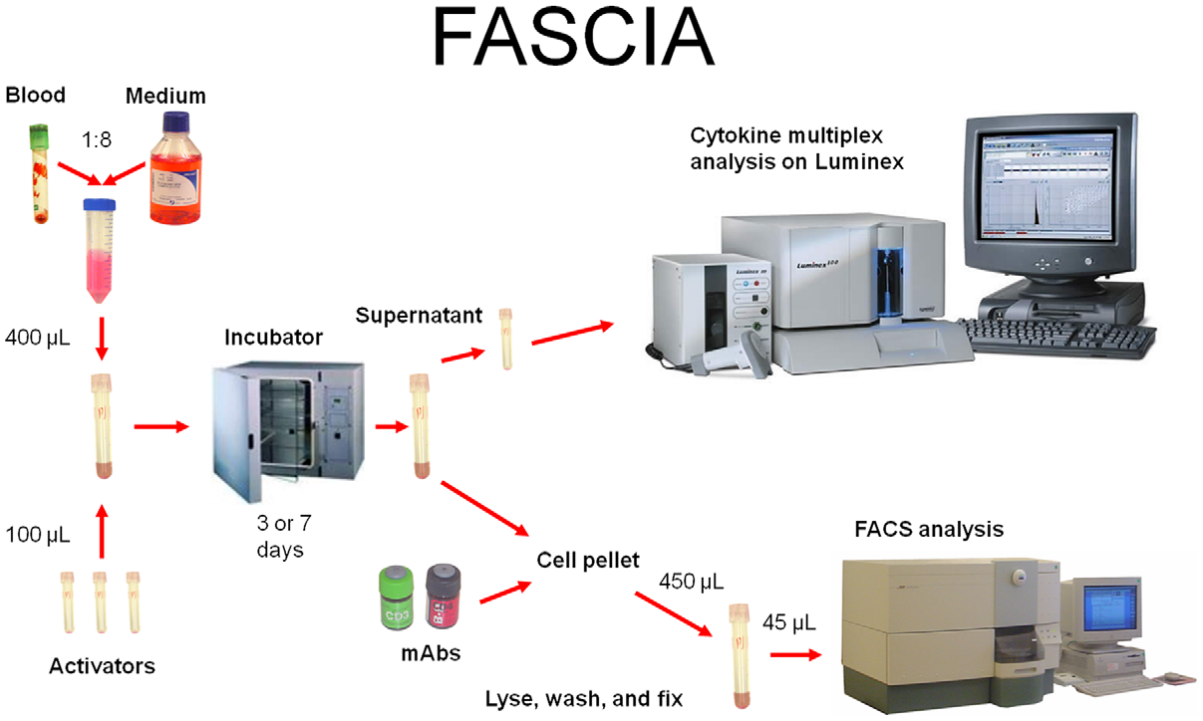
Schematic outline of the analysis of proliferative responses and cytokine production by FASCIA and Luminex assay.

The FASCIA was performed as earlier described [23], [24], [25], [26], [31] In short, whole-blood is cultured together with antigens of interest for 3 or 7 days, to develop peak levels of the particular immune-responses. Results are measured by flow cytometry detecting proliferative responses by enumerating lymphoblasts. Cytokine production or profiles assessing concentrations of cytokines in supernatants were measured by employing the Luminex instrument which allows detection of multiple cytokines simultaneously. A schematic outline of the procedure is given (Figure 1).

#### Whole-blood cell culture

Whole blood collected in vacutainer sodium heparine tubes was diluted 1∶8 in RPMI 1640 medium with GlutaMAX TM-1 61870 (Gibco, Invitrogen, UK) supplemented with 50 IU/ml penicillin (Gibco, Invitrogen, UK) and 50 ug/ml streptomycin (Gibco, Invitrogen, UK). 400 μl of the diluted blood and 100 μl of antigen or medium only were added to 12×75 mm polystyrene round-bottom tubes with caps (Falcon 352054, Becton Dickinson Labware, NJ), two tubes for each antigen. One set was incubated for 3 days and after incubation the supernatants were harvested and stored in −80°C for subsequent cytokine analysis. Cells from the three day culture were discarded. The other set was incubated for seven days. Supernatants were harvested and stored for cytokine analysis as described above and cell pellets were prepared for reading on Fluorescence Activated Cell Sorting (FACS. All incubations were carried out in a humidified atmosphere at +37°C with 7.5% CO2 in air. Caps were on the tubes but not tightened. Negative (medium only), positive phytohemagglutinin (PHA) (5 μg/ml) and Tuberculin (5 μg/ml, PPD RT 23, Statens Serum Institut, Copenhagen, Denmark) control cultures were run in parallel with specific stimulations ESAT-6 (2 μg/ml) and CFP-10 (2 μg/ml). From Statens Serum Institut we received a peptide pool of ESAT-6 defined by the following overlapping ESAT-6 peptides: ESAT-6^1−20^, ESAT-6^10−25^, ESAT-6^16−40^, ESAT-6^31−55^, ESAT-6^46−70^, ESAT-6^61−85^ and ESAT-6^71−95^, and a CFP-10 peptide pool defined by the overlapping CFP10 peptides CFP10^1−25^, CFP10^16−40^, CFP10^30−55^, CFP10^46−70^, CFP10^61−85^ and CFP10^76−100^ (SSI).

#### Proliferative response

The pellets were stained with 5 μl anti-CD3-Fitc and 5ul anti-CD4 PerCP [Becton Dickinson Immunocytometry Systems (BDIS), Stockholm, Sweden] for 15 min at room temperature. A lysing solution (1.0 ml Pharmlyse, BDIS) was added for 10 min at room temperature, followed by centrifugation, removal of the supernatants. Cells were lysed again with 0.5 ml Pharmlyse for 5 minutes. Then 1 ml Facsflow (BDIS) was added before centrifugation. Supernatants were removed and cells were fixed by resuspending them in 450 ul Cellfix (BDIS). The samples were stored in the dark at +4°C, until analyses on a FACScan or a FACScalibur (BDIS) using CellQuest software (BDIS), were performed. The instrument was calibrated to acquire 60±6 ul per minute and set for four-colour analysis using FACSComp software (BDIS) in conjunction with CaliBRITE (BDIS). 10% of the sample was acquired and saved as list-mode data for analysis, using a previously described method [23], [24]. The net result, with the number of proliferating lymphoblasts/ul blood, was determined by data analysis on Cell Quest Pro software (BDIS) after subtraction of negative (medium only) stimulations.

#### Cytokine and chemokine detection by multiplex technology

Bio-Plex cytokine reagent kits were purchased from Bio-Rad Laboratories, Hercules, CA, USA. The culture supernatants from day 3 were analysed for the following cytokines including limits of detection: IL-2 (1.3–27914 pg/ml), IP-10 (2.7–41761 pg/ml), TNF-α (5.7–76049 pg/ml) and IFN-α2 (2.05–1270 pg/ml). The culture supernatants from day 7 were analysed for; IL-4 (0.3–5334 pg/ml), IL-5 (2.3–9296 pg/ml), IL-6 (1.9–26814 pg/ml), IL-10 (1.7–7016 pg/ml), IL-13 (2.6–40302 pg/ml), IL-17 (1.9–32159 pg/ml), GM-CSF (0.8–11346 pg/ml), MIP-1b (1.1–3844 pg/ml). The incubation times that are optimal for each cytokine peak after specific antigen stimulation have been determined previously in our laboratory [25].

The cytokines were measured simultaneously using a multiplex assay based on xMAP technology developed by Luminex Corporation. The assay was performed according to the manufacturer's instructions with minor modifications.

Kit cytokine standard was dissolved and diluted in matrix solution consisting of RPMI medium supplemented with 10% volume/volume of the Serum Standard Diluent included in the kit. This matrix was also used as assay blank and to dilute the in-house control. Preparation was performed in 96-well microtitre filter bottom plate and all washes were done by vacuum filtration using an Aurum Vacuum Manifold from Bio-Rad. All incubations were performed at room temperature on an orbital shaker.

Briefly the preparation protocol was carried out as follows. 50 ul of mixed beads were added to the prewet wells and washed two times. After addition of 50 ul of standard, in-house control or sample the plate was incubated for one hour. Samples were added undiluted and no dilution factor was used in results calculations. After the subsequent washing 25 µl of detection antibody mixture were added to each well and the plate was incubated for 30 min and then washed. 50 ul of streptavidin-PE were added and after 10 min incubation the plate was washed and the beads were finally re-suspended in 125 ml of assay buffer. Assay readings were carried out using the Luminex 100 platform (Bio-Rad Laboratories) with Bio-Plex manager software (Bio-Rad version 5.0) at low target reporter calibration. Sample size was set to 50 ul and data acquisition was 50 beads per region. Standard curves were analysed using a Five- Parameter Logistic (5PL) regression curve fitting (Bio-plex software). Sample concentrations were interpolated from the standard curves.

To validate the method we used an in-house control constituting aliquots of PHA stimulated whole blood culture supernatant. This control was used to evaluate the inter-assay variability throughout the series of runs. To cover different parts of the standard curve the in-house control was used both undiluted and diluted 1∶25. With the undiluted control coefficient of variation (CV) was below 27% for all cytokines within standard range. CV for control diluted 1∶25 was below 11% for all cytokines within standard range, except MIP-1beta which had a CV of 54.7%.

Un-stimulated cytokine responses were subtracted from antigen stimulated results. The cut-offs were set high to get 100% specificity in this material. The highest values among the TB negative controls plus 0.1 pg/ml were used for each biomarker.

### 4 Radiology

Two radiologists, not blinded to clinical information, read the radiographs independently since this was a study in a clinical setting and the definition of active TB was based on microbiological rather than radiographic criteria.

Apical involvement, cavities with acinonodular foci, miliary pattern, localized small fibronodular foci, unilateral hilar and/or mediastinal adenopathy with or without localized alveolar opacities and pleural effusion were interpreted as radiographic changes suggestive of active TB.

Solitary calcified nodules were interpreted as radiographic changes suggestive of previous primary TB infection.

Previously active but healed TB was suspected when there was fibrotic scarring of the upper lung lobes and loss of lung volume as well as apical pleural scarring.

### 5 Statistical methods

Comparisons of cytokines in terms of positive/negative results were made with McNemar`s test. The Kruskal Wallis test for nonparametric data was performed for the comparison of cytokine levels in all subgroups and Wilcoxon's rank sum test was used as a post-hoc test for finding where the differences laid. P values <0.05 were considered significant. Ninety-five percent confidence levels (Cl) for shift in median vales were calculated using Hodges Lehman estimation. Spearman's rank correlation coefficient was used for comparison of cellular responses to cytokine levels; levels above 0.7 were considered well correlated. Confidence levels were calculated using Fisher's Z-transform. Heat mapping of cytokine results was performed with the statistical program Rv.2.14, using the package g-plots. Actual cytokine levels were logged, and then measured in times higher than cut-off. The quota for each cytokine was then sorted by proliferative responses in each row and marked with a colour-code to create the heat map image.

Analyses were conducted using SAS v.9.2.

## Results

### 1 Patients

Forty-nine patients (58%) were women, 36 (42%) were men. Their nationalities represented all continents except Australia. Forty-six patients (54%) came from TB high-endemic areas (defined as TB incidence ≥100/100 000) and the largest groups of patients came from Somalia (n = 17) and Sweden (n = 17).

Of the 54 patients with active TB, 33 (61%) had PTB, including 2 with dissemination. Twenty-one (39%) patients had EPTB with manifestations of lymph node TB (n = 13), gastrointestinal TB (n = 1), urogenital TB (n = 2), skeletal TB (n = 4), and ocular TB (n = 1). Active TB was verified by mycobacterial culture in 24/33 (73%) PTB cases and 14/21(67%) EPTB cases and by *Mtb complex* PCR only in 1/33 PTB cases and 1/21 EPTB cases. Three EPTB patients were verified by histopathological diagnosis of biopsy material from lymph nodes indicating granulomatous lymphadenitis. The remaining 8 PTB cases were diagnosed by suggestive radiology, clinical symptoms and response to TB treatment and the remaining 3 EPTB cases were diagnosed by clinical response to treatment (clinical TB) (Table 1).

The 8 cases with no active TB were diagnosed with a psoatic abscess of common bacterial origin (n = 1), sarcoidosis (n = 1), bacterial pneumonia (n = 3), erythema nodosum (n = 1), fibroma molle (n = 1) and bronchiectasias (n = 1).

### 3 Cytokine results

After having chosen a cut-off for each cytokine, we could analyse the nature of cytokine responses to CFP-10 and ESAT-6 (Table 2). The FASCIA results from parallel negative and positive control cultures were used as a quality control to decide whether each subjects corresponding culture supernatant would be included in multiplex analysis or not. All samples were accepted. The limit of detection for each cytokine is presented in the Methods section.

**Table 2:** Cytokine results from multiplex analysis and proliferative responses from FASCIA after CFP-10 and ESAT-6 stimulation in patients with active-, latent- and non tuberculosis.

	Active tuberculosis (ATBI)	Non Tuberculosis	Cut-off	Sensitivity ATBI		Latent Tuberculosis (LTBI)	Sensitivity LTBI
FASCIA-positive proportion (%)	42/54 (77%)	0/8 (0%)				11/23 (48%)	
**CFP-10**	Median (IQR)	Median (IQR)	Threshold (pg/ml)	Antigen Separate	Antigens Combined	Median (IQR)	%
CD3+CD4+cells/µl	52 (3.7–227.8)	1 (0.75–2.3)				4.0 (0–11.5)	
MIP-1beta	103,7 (35.7–328.9)	0 (0–8.5)	84	56	91	0 (0–9.2)	17
IFN-gamma	35.5 (2.2–296.9)	0 (0–0)	5.9	65	84	0 (0–5.9)	26
GM-CSF	2.5 (0–16.5)	0 (0–0)	0.1	63	79	0 (0–0.3)	26
IL-6	68.5 (22.8–519.9)	8.5 (5.9–24.6)	82.1	51	63	7.6 (3.6–11.4)	4
IL-13	0 (0–12.9)	0 (0–0)	0.1	49	63	0 (0–0)	13
IL-5	0 (0–3.3)	0 (0–0)	0.1	30	42	0 (0–0)	9
IL-4	0 (0–0)	0 (0–0)	0.1	14	23	0 (0–0)	4
IL-10	0 (0–1.8)	0 (0–0)	2.3	16	33	0 (0–0)	4
IL-12	0 (0–0)	0 (0–0)	0.1	0	0	0 (0–0)	0
IL-17	0 (0–0)	0 (0–0)	0.1	9	16	0 (0–0)	4
IL-2	16.3 (5.8–47.9)	0 (0–0)	1.5	84	93	0 (0–0)	17
IP-10	1438.5 (367.2–2455.3)	19.3 (6.4–29.7)	100	93	100*	120.3 (84.4–214.4)	70
TNF-alpha	17.6 (5.9–64.0)	0 (0–0)	0.1	79	81	0 (0–6.3)	30
IFN-alpha2	0 (0–0.6)	0 (0–0)	0.1	26	33	0 (0–0)	22
**ESAT-6**							
CD3+CD4+cells/µl	91 (6–355.0)	2.0 (0–2.1)				3.0 (1.0–8.0)	
MIP-1beta	137.8 (63.7–350.9)	0 (0–8.0)	35.4	91	91	0 (0–16.6)	22
IFN-gamma	139.2 (5.4–536.8)	0 (0–0)	7.1	74	84	0 (0–2.0)	17
GM-CSF	8.7 (0–33.0)	0 (0–0)	0.1	72	79	0 (0–0.8)	30
IL-6	197.9 (58.0–547.4)	10.3 (5.1–23.2)	147.4	53	63	10.2 (6.2–33.2)	17
IL-13	5.0 (0–21.0)	0 (0–0)	0.1	60	63	0 (0–0)	22
IL-5	0 (0–5.1)	0 (0–0)	0.1	33	42	0 (0–0)	17
IL-4	0 (0–0)	0 (0–0)	0.1	19	23	0 (0–0)	9
IL-10	0 (0–2.2)	0 (0–0)	2.1	26	33	0 (0–0)	9
IL-12	0 (0–0)	0 (0–0)	0.1	0	0	0 (0–0)	0
IL-17	0 (0–0)	0 (0–0)	0.1	12	16	0 (0–0)	13
IL-2	21.9 (4.8–50.5)	0 (0–0)	2.1	79	93	0 (0–2.6)	30
IP-10	983.2 (484.4–2477.6)	25.7 (7.2–47.3)	72.2	100	100*	158.2 (90.5–291.6)	87
TNF-alpha	30.9 (0–160.2)	0 (0–0)	0.1	72	81	0 (0–10.1)	30
IFN-alpha2	0 (0–0)	0 (0–0)	0.1	23	33	0 (0–0)	4

Proportion of subjects with positive FASCIA is expressed as n/total for each group. Cut-off thresholds were set to gain 100% specificity for all assays. Each assay's sensitivity for active tuberculosis is calculated from these cut-offs. Sensitivity is shown for each antigen separate and when both are used in combination. Cytokine median results are presented in units of pg/ml and shown together with interquartile range (IQR). *IP-10 is significantly more sensitive than IFN-γ as a marker for active verified TB (p = 0.0233).

### *Active TB versus controls*

Compared to controls, the cytokines IFN-γ, IP-10, IL-2, TNF-α, MIP-1β, GM-CSF, IL-13 and IL-6 were produced significantly more often in higher amounts (p<0.05 with the Kruskal Wallis and Wilcoxon's rank sum test) in samples from patients with active TB after stimulation with ESAT-6 and CFP-10 (Table 3). Specific p-values and confidence levels are presented in Table 3. Some production of cytokines was detected in the controls, but the median level and the 75% percentile were well below the chosen cut-offs for each cytokine (Table 2).

**Table 3:** Significant median differences (MD), confidence intervals (CI) and P-values (p) for cytokine levels in active TB compared to controls and LTBI after stimulation with CFP-10 and ESAT-6.

	Active TB vs controls				Active TB vs LTBI			
	CFP-10		ESAT-6		CFP-10		ESAT-6	
Cytokine	MD (CI)	p	MD (CI)	p	MD (CI)	p	MD (CI)	p
IFN-γ	36 (6–216)	<0.0001	139 (12–289)	<0.0001	33(2–120)	<0.0005	29 (2–190)	0.0004
IP-10	1357(411–2076)	<0.0001	920(531–1740)	<0.0001	688(320–1618)	<0.0001	741(303–1230)	<0.0001
IL-6	182 (59–364)	0.0017	182 (59–364)	<0.0001	40 (18–194)	0.0002	125 (42–270)	0.0002
IL-2	16 (9–41)	<0.0001	22 (6–42)	<0.0001	13(7–36)	<0.0001	13 (2–29)	0.0008
TNF-α	18 (10–54)	<0.0001	31 (9–104)	<0.0001	16 (6–38)	0.0007	19 (0–85)	0.002
MIP-1β	57 (4–178)	<0.0001	91 (38–257)	<0.0001	78 (36–152)	<0.0001	93 (61–243)	<0.0001
GM-CSF	2 (0–9)	0.0002	9 (1–16)	<0.0001	1 (0–6)	0.0088	3 (0–13)	0.0062
IL-13	0 (0–9)	NS	5 (0–14)	0.0001	0 (0–9)	NS	3 (0–12)	0.0071

NS: non significant.

**Figure 2:**
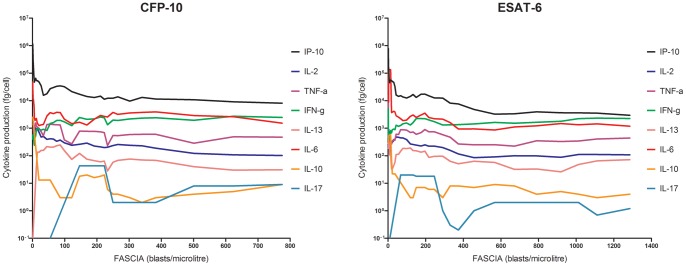
Cytokine results from patients with verified active TB, after stimulation with CFP-10 (2 A) and ESAT-6 (2 B), in relation to cell proliferation. The data are presented as graphs with the number of lymphoblasts/μl on the X-axis and the level of cytokine production/cell (fg/cell) on the Y-axis. These graphs show lower levels of cytokines produced per cell, in strong responses, except for IFN-γ, where levels rise accordingly, although a few outliers influence ESAT-6 results for low levels of lymphoblasts/µl.

In patients with active TB, we found a high correlation, using Spearman's rank correlation coefficient, between the number of cells detected and the corresponding cytokine levels after stimulation with either CFP-10 or ESAT-6 (Table 4). Figure 2A (CFP-10) and 2B (ESAT-6) illustrate the production of IFN-γ, IP-10, IL-2, TNF-α, IL-6, IL-10, IL-13 and IL-17 in relation to cell numbers.

**Figure 3:**
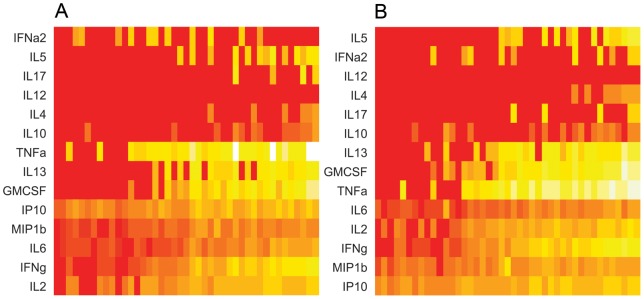
Heat maps: Cytokine levels in verified active TB patients divided by cut-off levels, after stimulation with CFP-10 (3 A) and ESAT-6 (3 B). The individual values are represented as colours. Light colours indicate high levels of cytokines and vice versa. On the Y-axis: The different cytokines tested with the FASCIA. On the X-axis: The cytokine levels, divided by the cut-off level for each cytokine, logged and sorted by rising proliferative responses (the higher the proliferative response, the further to the right).

**Table 4:** Correlation coefficients (CC) and confidence intervals (CI) with 95% confidence levels for the comparison of cellproliferation numbers and cytokine levels in patients with active TB.

Cytokine	CFP-10	ESAT-6
	CC (CI)	CC (CI)
IFN-γ	0.95 (0.91–0.97)	0.95 (0.91–0.97)
IL-2	0.90 (0.82–0.94)	0.90 (0.83–0.95)
TNF-α	0.90 (0.81–0.94)	0.90 (0.82–0.94)
GM-CSF	0.90 (0.82–0.94)	0.90 (0.89–0.97)
IL-6	0.88 (0.78–0.93)	0.79 (0.63–0.88)
IP-10	0.80 (0.65–0.89)	0.80 (0.65–0.88)
MIP-1β	0.81 (0.66–0.89)	0.77 (0.60–0.87)
IL-13	0.76 (0.59–0.86)	0.81 (0.68–0.89)

IP-10 was the only biomarker detected in samples with cell-numbers below the cut-off level for the proliferative response (6cells/μl). IP-10 was positive in all patients with microbiologically confirmed TB, after stimulation with ESAT-6 (Fig 2B). IP-10 was the only marker to be significantly more sensitive in diagnosing microbiologically verified TB compared to IFN-γ (p = 0.0233 McNemar's test). The highest sensitivities for active TB were found in IP-10, IL-2, MIP-1β, IFN-γ and TNF-α, all above 80% with the combination of both antigens (figure 3A (CFP-10) and 3B (ESAT-6) and Table 2, column “Antigens combined”).

IFN-γ, IL-2, MIP-1β and TNF-α were detected above the threshold level (>6 cells/μl) in patients with active TB. GM-CSF, IL-5, IL-6 and IL-13 were detectable at >50 cells/μl. IL-10 and IL-17 were often detectable at >500 cells/μl, while IL-12 and IL-4 were never detected even though the cell proliferation was >1000 cells/μl. IFN-α2 was detected in a sporadic way and did not correlate to cell proliferation numbers. The results were similar after stimulation with both antigens (CFP-10 and ESAT-6). Thus, samples with cellular numbers below 50 cells/μl showed a Th1 weighted cytokine response and samples with cellular numbers above 50 cells/μl had both a Th1 and a Th 2 cytokine response. Samples with >50 cells/μl more often derived from patients with EPTB (59% for CFP-10 and 54% for ESAT-6). See Figure 2.

#### PTB versus EPTB

After stimulation with ESAT-6, only two cytokines, IL-2 (p = 0.046) and IL-13 (p = 0.037), differed significantly between EPTB (highest response) and PTB. The differences of median values were 18 pg/μl (0–44) for IL-2 and 11 pg/μl (0–28) for IL-13. After stimulation with CFP-10 there was no significant difference between these groups.

#### Immunosuppressed TB patients

There was no significant difference in cytokine levels in immunosuppressed TB patients with active TB for any of the antigens included in our study, compared to immune-competent patients (Fig. 2). Three pregnant and thereby potentially immune-suppressed patients, one patient with rheumatoid arthritis and one HIV patient responded well in multiplex analysis, similar to the cytokine patterns of non-immune-suppressed patients. Three patients, one with diabetes mellitus, one with kidney failure and one with HIV, responded with lower levels of the Th1- pro-inflammatory cytokines/chemokines, and a liver transplanted patient only responded with IP-10 (175 pg/μl) and IL-2 (6 pg/μl), just above each cut-off, after ESAT-6 stimulation. The sensitivity of IP-10 among immune-suppressed verified TB-patients was 9/9 (100%).

#### LTBI versus active TB

The pattern of cytokines was similar in samples from patients with active TB and in patients with LTBI (as defined by TST), but the cellular responses were much more frequent in active TB and with higher levels of cytokines. These levels were significant for IFN-γ, IP-10, MIP-1β, IL-2, TNF-α, IL-6 and GM-CSF after stimulation with both ESAT-6 and CFP-10, compared to patients with LTBI and for IL-13 after stimulation with ESAT-6 (p<0.05) (s Table 2 and Table 3).

However, five patients in the LTBI group also had very high levels of IP-10, TNF-α and IFN-γ. In a clinical follow-up, two patients were lost to follow-up and the other three had no symptoms of active TB disease after a follow-up of approximately two years. IP-10 after stimulation with ESAT-6 in these patients gave the most frequent reactivity (87%). IP-10 after stimulation with tuberculin was positive for all LTBI patients.

#### Clinical TB

The cytokine/chemokine responses in the group of clinical TB (n = 11) were less frequent, showing negative results for several cytokines in three patients. The cytokine levels in the positive responders were similar to those found in the group of verified TB. IP-10 detected 10/11 (91%) cases after stimulation with ESAT-6, which is higher than the frequency of cellular responses 5/11 (45%). IL-2 detected 6/11 (54%) with CFP-10 or ESAT-6 and IFN-γ, MIP-1β, TNF-α and IFN-α2 detected 5/11 (45%) with either antigen.

## Discussion

### Active TB

In the present study, Th1-proinflammatory cytokines were expressed at high levels with both CFP-10 and ESAT-6 stimulation in samples from patients with microbiologically/histopathologically verified active TB and the results correlated very well with proliferative responses. In particular, production of IP-10, IFN-γ, IL-2, MIP-1β and TNF-α was detected in these patient samples, consistent with previous studies [20]. The proliferative responses confirm that the Th1-cytokines are mainly derived from CD4+ cells.

In a study by Dlugovitzky et al, the Th1 responses appeared stronger in patients with mild to moderate disease compared to patients with severe disease [32], which is similar to our results, with higher cell proliferation and cytokine production (IL-2) in patients with EPTB with milder, mainly localized, TB manifestations, and lower levels in more severely diseased and thereby potentially immune-suppressed PTB patients.

Th 2 derived cytokines (IL-13 and IL-5) were detected in patients with active TB with higher proliferative responses (>50 cells/μl). These proliferative responses were more often found in EPTB patients, than PTB patients. IL-13 was significantly higher in the EPTB group, which in our study mainly consisted of localized lymph node TB but also some cases of disseminated TB disease, skeletal and ocular TB.

IL-10 and IL-17 were also detected in the verified TB patients but only in those with proliferative responses >500 cells/μl, which could be explained by the multiplex not being sufficiently sensitive to detect such low levels of IL-10 and IL-17 responses. These results differ from those in the study by Sutherland et al [20], where no IL-17 responses were detected at all. Th-17 cells have been suggested to play a role in human TB immunity through the recruitment of neutrophils in granuloma-formation, thus functioning as a bridge between innate and adaptive immunity [33]. Th-17 knockout mice show inability to form granuloma [34] but the dispensability of these cells in human infection still remains unclear.

Our IL-10 results indicate a T regulatory activity in patients with active TB; still higher levels could possibly have been found at the site of infection compared with peripheral blood [35], [36], [37].

Since IFN-α2 is a macrophage-derived substance, it is not surprising that its responses in this study were completely uncorrelated to CD4+ cell responses in patients with active TB. Although levels of this cytokine were high in some cases, it was frequently undetected in *M tb* infection and therefore, according to this study, not useful for TB diagnosis.

GM-CSF was detected in patients with active TB, with levels high above cut-off. This cytokine also seems to be a promising biomarker, and its role in *Mtb* immunity is being further investigated in an ongoing study of PTB patients' contacts.

### IP-10 in active and latent TB

IP-10 was positive in all but one of the patients with verified active TB, which gave a sensitivity of 97%. This is high compared to previous studies, were sensitivities of 83% and 81% were found in active TB (verified by culture, PCR, microscopy or typical histology) by short-term stimulation multiplex assay [16], [17] and 75% by IP-10 ELISA [14] in HIV+ patients with active TB verified by sputum culture or microscopy. Another study of IP-10 performance in active culture-verified TB patients (91.5%) and TB suspicious radiology (8.5%) by EIA after overnight incubation with QuantiFERON antigens showed a 92.5% sensitivity of IP-10 but specificity was poor (48%), compared to healthy adults [38].

Cut-off for IP-10 was set at a high level in this study to reach 100% specificity but further evaluation is needed in further studies to set the final cut-off.

The median values of IP-10 in active TB in our study (HIV incidence <5%) were also considerably higher than in another study with analysis by an unstimulated ELISA assay in HIV seronegative patients with active TB [28], [39]. The lower sensitivity of IP-10 in previous studies could be due to the shorter incubation time with specific TB antigens for 18–24 h in the studies by Ruhwald, Goletti and others [14], [16], [17], [40], [41] whereas in our study stimulation continued for 3 days, the optimal time point for IP-10 detection.

IP-10 is produced by monocytes-macrophages after IFN-γ and TNF-α stimulation and induces chemotaxis on monocytes [42] and lymphocytes in the formation of granulomas. IP-10 is also part of the innate immunity where it is produced by fibroblasts and acts as an antimicrobial peptide against extracellular bacteria [43]. IP-10 has also been proposed as a nonspecific marker, measured un-stimulated by a commercial ELISA, for monitoring disease activity in *Mtb* infection [39].

Our results concerning IP-10 seem to add up to this chemokine being an important factor and an aid in the diagnosis of difficult-to-diagnose cases of active TB, e.g. in immuno-suppressed patients with diffuse symptoms and negative microbiological testing. It has also been suggested that IP-10 may be used to monitor therapy effect in experimental tests [44].

A limitation in our study is that sensitivity and specificity were estimated on the same groups that were used to establish the cut-offs; the differences in cytokine levels between positive and negative samples need further evaluation.

IP-10 levels were significantly higher in patients with active TB than in LTBI, but 5/23 (22%) patients in the latter group also had unexplained high levels. These patients have not progressed to active TB as far as we know.

The results concerning different levels of IP-10 production in active TB and LTBI need further study. This is being explored in an ongoing study of a cohort of contacts to patients with microbiologically verified PTB.

### Active verified TB versus LTBI

Significant differences were found between the groups of active TB and LTBI for several biomarkers. These results must be interpreted with caution, as our study was not designed to define LTBI patients beyond having a positive tuberculin test and an origin from a TB endemic area. However, the predictive value of a positive TST was judged to be high in our patients with presumed LTBI [45]–[46], as the majority had been BCG-vaccinated only once in infancy, but whether a positive TST reflects a true LTBI or a mere immunological memory remains unclear.

Interestingly enough, cytokine patterns formed were similar in active TB and LTBI, with dominant Th1- pro-inflammatory cytokines detected. Only in cases with high cellular proliferative responses did we detect Th2-, Th17- and T- regulatory biomarkers.

### Clinical TB

This group of culture-, sputum-microscopy and PCR negative TB patients gave less frequent responses with the chosen biomarkers than verified TB patients, but 91% of these cases were positive for IP-10 after stimulation with ESAT-6. It may, of course, be questioned whether these patients really were symptomatic due to TB rather than being latently infected, never *Mtb* infected at all and/or had e.g. a concomitant common bacterial infection. All of them responded to TB chemotherapy, which also patients with a common bacterial infection could have done. Sputum-negative PTB patients and patients with EPTB in high TB endemic areas are difficult to diagnose and therefore constitute a “high priority area of research” [47]. In this context IP-10 seems to be a potentially important biomarker. The question is whether IP-10 is sufficiently specific to differentiate between the clinical entities of active smear-negative TB, EPTB and LTBI, which is a common dilemma also in our setting with TB suspects mainly originating from TB high endemic areas.

### Conclusions

The Th1-derived cytokine IP-10 after *FASCIA* and multiplex analysis is an interesting biomarker, as are the other Th1- cytokines, with very high sensitivities in *Mtb* infection, and a high specificity compared to controls with no exposure to *Mtb*. They could possibly be used, after setting adequate cut-offs for active TB and LTBI, to differentiate between different clinical entities of *Mtb* infection. Th2-, Th17- and T- regulatory biomarkers could only be detected in patients with high cellular proliferation, more often EPTB patients, predominantly consisting of lymph node TB, but also disseminated disease, in our study.

Our results need further studies, including new latency antigens, in larger patient groups of LTBI [48], where the clinical need of better diagnostic methods is immense.
